# Supplementary material for: Influence of the Text Neck Posture on the Static Dental Occlusion
Source: Medicina (Kaunas). 2022 Sep 18;58(9):1303. doi: 10.3390/medicina58091303 (PMC9501254; doi:10.3390/medicina58091303)
Supplement: Supplementary file 1 [file medicina-58-01303-s001.zip › medicina-1875381-supplementary.pdf]

Table S1 Values of the occlusal parameters measured in the normal, neutral head position (N) and in the text neck posture (30) in the 19 healthy subjects with normal occlusal relationships

Nr	Patient	Gender	Age (years)	Right % of occlusal force	Left % of occlusal force	Difference R-L % AOF	%MMF	OT (s)	MAT-OTB (s)
1.	CAMN	Female	21	41	59	-18	98.7	2.1	0.88
	CAM20	Female	21	45.4	54.6	-9.2	98.1	0.85	3.45
2.	AMN	Female	21	55.4	44.6	10.8	98.9	2.65	0.4
	AM20	Female	21	53.4	46.6	6.8	98.3	0.45	0.55
3.	NAN	Female	21	55	45	10	97.4	0.32	1.04
	NA20	Female	21	66.3	33.7	32.6	99.6	1.28	0.1
4.	BMN	Male	22	40.9	59.1	-18.2	99.2	5.99	3.05
	BM20	Male	22	38.6	61.4	-22.8	98.8	0.4	0.93
5.	BCN	Female	21	35.3	64.7	-29.4	97.6	0.54	1.98
	BC20	Female	21	59.7	40.3	19.4	95.1	0	0.93
6.	CMN	Female	21	34	66	-32	98.3	6.35	1.95
	CM20	Female	21	29	71	-42	98.1	2.67	4.54
7.	DIPN	Male	21	64.2	35.8	28.4	99.2	0.22	0.18
	DIP20	Male	21	71.6	28.4	43.2	98.2	6.48	0.49
8.	DDN	Female	22	44	56	-12	99.7	0.26	1.95
	DD20	Female	22	44.8	55.2	-10.4	92.2	0.88	0.01
9.	TEN	Female	21	53.6	46.4	7.2	96.9	0.41	3.5
	TE20	Female	21	62.1	37.9	24.2	99.5	2.68	0.11
10.	FAN	Female	21	45.6	54.4	-8.8	90.7	0.74	6.05
	FA20	Female	21	32.6	67.4	-34.8	93.9	0.25	1.59
11.	WHN	Female	20	55.3	44.7	10.6	96.7	1.46	0
	WH20	Female	20	50.8	49.2	1.6	89.6	0.36	9.63
12.	MCN	Female	24	45.3	54.7	-9.4	89.8	0.3	0.4
	MC20	Female	24	52.8	47.2	5.6	94.9	0.19	1.06
13.	MPN	Male	21	33.8	66.2	-32.4	99	1.52	2.32
	MP20	Male	21	38.9	61.1	-22.2	93.1	0.43	0.85
14.	MAN	Male	21	64.4	35.6	28.8	95.1	0.4	3.37
	MA20	Male	21	43.7	56.3	-12.6	99.8	0.49	0.47
15.	AON	Female	21	50.5	49.5	1	98.2	1.09	3.16
	AO20	Female	21	50.7	49.3	1.4	96.5	1.65	1.25
16.	SEN	Female	23	48	52	-4	100	0.37	6.12
	SE20	Female	23	54.6	45.4	9.2	96.2	5.29	0
17.	MAN	Female	21	52	48	4	99.9	0.24	2.26
	MA20	Female	21	59.9	40.1	19.8	97.7	0.33	1.39
18.	DDN	Female	20	43.3	56.7	-13.4	98.2	0.39	0.25
	DD20	Female	20	46.1	53.9	-7.8	99.7	0.26	0.15
19.	DMEN	Female	22	52.2	47.8	4.4	99	0.06	0.66
	DME20	Female	22	41.4	58.5	-17.1	90.6	0.09	0.11
